# Scaling up efforts to target evolving viruses

**DOI:** 10.7554/eLife.110630

**Published:** 2026-02-23

**Authors:** Lin Wang, Henrik Salje

**Affiliations:** 1 Department of Genetics, University of Cambridge Cambridge United Kingdom

**Keywords:** influenza, sequencing-based neutralization assay, virus evolution, antigenic drift, vaccine, H3N2, Viruses

## Abstract

High-throughput neutralisation tests could lead to a better understanding of the evolution of human influenza.

**Related research article** Kikawa C, Loes AN, Huddleston J, Figgins MD, Steinberg P, Griffiths T, Drapeau EM, Peck H, Barr IG, Englund JA, Hensley SE, Bedford T, Bloom JD. 2025. High-throughput neutralization measurements correlate strongly with evolutionary success of human influenza strains. *eLife* **14**:RP106811. doi: 10.7554/eLife.106811.

The ability of viruses and other pathogens to mutate and evolve into new strains has long been a challenge for the immune system and for researchers designing vaccines. It has also become clear over the past decade that the immune response to a specific strain can influence the risk of disease following subsequent exposures (Koel et al., 2013; Wang et al., 2024). However, our understanding of the breadth and specificity of immune responses remains limited. This knowledge gap limits our ability to understand how immunity to pathogens such as influenza, dengue, or SARS-CoV-2 is evolving as new strains emerge, as well as our ability to design vaccines that are targeted at the strains that present the greatest risks.

A key barrier in gaining detailed insight into an individual’s ability to neutralise the different strains that circulate within a population is the cumbersome nature of most functional assays. Neutralisation tests, such as plaque reduction neutralisation tests, require serial dilutions of an individual’s blood sample to be incubated in wells where a single viral strain has been added (Russell et al., 1967). Neutralisation titres – which measure the ability of antibodies in the sample to reduce cells infected by a specific strain – are then estimated by counting the number of plaques formed in each well and comparing these across different dilutions. Determining the titre for a single viral isolate demands multiple wells and skilled technicians, making it labour-intensive. Furthermore, variability in titres across laboratories can be significant, depending on the specific protocol used (Thomas et al., 2009). Ultimately, these limitations mean that few laboratories perform large-scale plaque reduction neutralisation tests.

High-throughput assays of blood samples are capable of capturing antibodies that are bound to pathogen-derived proteins (Chan et al., 2022; Mohan et al., 2018). Assays like Luminex-based multiplex assays and PhIP-seq platforms mainly focus on identifying which pathogen(s) have infected an individual or circulated within a community. However, these assays are less well suited to capturing the diversity of host immune responses to a single pathogen. Moreover, they only measure the ability of antibodies to bind to a pathogen – they cannot tell us if the antibodies are able to neutralise (i.e., kill) the pathogen.

Now, in eLife, Jesse Bloom and colleagues – including Caroline Kikawa and Andrea Loes as joint first authors – report that they have developed a sequencing-based method in which neutralisation titres are measured for each individual’s serum against multiple influenza strains at the same time (Kikawa et al., 2025). This new approach enabled the researchers to test hundreds of serum-virus pairs within a single 96-well plate. In contrast, traditional plaque reduction neutralisation tests typically test only 8–12 pairs per plate.

Kikawa et al. – who are based at the Fred Hutch Cancer Center in Seattle, the University of Washington, and other institutions in Seattle, Philadelphia and Melbourne – applied this technology to 150 sera and 78 different H3N2 influenza viruses, producing over 11,000 neutralisation titres. The results revealed clear person-to-person variations in titres and showed that epidemiologically fitter strains were linked to weak population immunity. In other words, the lower the level of antibodies in the population against a given strain, the faster that strain spreads.

This technology has substantial potential. Each year, WHO Collaborating Centres on Influenza characterise tens of thousands of circulating influenza strains using haemagglutination inhibition (HAI) assays with ferret sera (Ziegler et al., 2022). A subset of these strains undergoes neutralisation testing with ferret or pooled human sera. These data, along with genetic analysis of sequenced isolates, inform the WHO’s recommendations for influenza vaccine composition. However, limitations in HAI testing capacity mean only a fraction of viral diversity can be considered, and there can be long delays between isolates being identified and their neutralisation profile being captured.

The technology developed by Kikawa et al. could substantially improve the range of viruses tested and efficacy of the platform, allowing for more viruses to be considered in a shorter time period. Moreover, it opens up possibilities for a more detailed understanding of immune signatures linked to infection risk at both the individual and the population levels, and for identifying specific viral changes associated with escape from natural infection or vaccine-derived immunity (Fonville et al., 2014). Finally, this approach could be applied to other pathogens, such as dengue virus, RSV, and coronaviruses, by combining the non-structural region of a reference genome with strain-specific structural proteins to generate strain-specific chimeric viral particles.
